# Rab27a‐dependent exosomes protect against cerebral ischemic injury by reducing endothelial oxidative stress and apoptosis

**DOI:** 10.1111/cns.13902

**Published:** 2022-06-29

**Authors:** Xiaotang Ma, Jia Zhao, Suqing Li, Yan Wang, Jinhua Liu, Yumeng Shi, Jiehong Liu, Yanyu Chen, Yanfang Chen, Qunwen Pan

**Affiliations:** ^1^ Guangdong Key Laboratory of Age‐related Cardiac and Cerebral Diseases, Institute of Neurology Affiliated Hospital of Guangdong Medical University Zhanjiang China; ^2^ Emergency Department Affiliated Hospital of Guangdong Medical University Zhanjiang China; ^3^ Institute of Biochemistry and Molecular Biology Guangdong Medical University Zhanjiang China; ^4^ Department of Pharmacology and Toxicology Boonshoft School of Medicine, Wright State University Dayton Ohio USA

**Keywords:** apoptosis, exosomes, ischemic stroke, oxidative stress, Rab27a

## Abstract

**Introduction:**

Multicellular crosstalk within the brain tissue has been suggested to play a critical role in maintaining cerebral vascular homeostasis. Exosomes (EXs) mediated cell–cell communication, but its role in cerebral ischemic injury is largely unknown. Rab27a is one of the major genes controlling EX release. Here, we explored the role of Rab27a in regulating brain EXs secretion, and the effects of Rab27a‐mediated EXs on ischemia evoked cerebral vascular disruption and brain injury.

**Methods:**

Cerebral ischemia was induced in Rab27a knockout (Rab27a^−/−^) and wide type (WT) mice by transient middle cerebral artery occlusion (tMCAO). Differential gene expression analysis was performed in ischemic brain tissue by using mRNA sequencing. EXs isolated from brain tissue of Rab27a^−/−^ and WT mice (EX^WT^ or EX^Rab27a−/−^) were pre‐administrated into tMCAO operated Rab27a^−/−^ mice or oxygen and glucose deprivation (OGD) treated primary brain vascular endothelial cells (ECs).

**Results:**

We demonstrated that Rab27a expression in the peri‐infarct area of brain was significantly elevated, which was associated with local elevation in EXs secretion. Rab27a deficiency dramatically decreased the level of EXs in brain tissue of normal and tMCAO‐treated mice, and Rab27a^−/−^ mice displayed an increase in infarct volume and NDS, and a decrease in cMVD and CBF following tMCAO. Pre‐infusion of EX^WT^ increased the brain EXs levels in the tMCAO operated Rab27a^−/−^ mice, accompanied with an increase in cMVD and CBF, and a decrease in infarct volume, NDS, ROS production, and apoptosis. The effects of EX^Rab27a−/−^ infusion were much diminished although in a dose‐dependent manner. In OGD‐treated ECs, EX^Rab27a−/−^ showed less effectivity than EX^WT^ in decreasing ROS overproduction and apoptosis, paralleling with down‐regulated expression of NOX2 and cleaved caspase‐3.

**Conclusion:**

Our study demonstrates that Rab27a controls brain EXs secretion and functions, contributing to cerebral vascular protection from ischemic insult by preventing oxidative stress and apoptosis via down‐regulating NOX2 and cleaved caspase‐3 expression.

## Graphical abstract

Ischemia promotes Rab27a dependent brain exosomes secretion, which is associated with the severity of ischemic injury. Rab27a dependent exosomes contribute to protect cerebral vessels from ischemic injury by preventing oxidative stress and apoptosis. The underlying mechanisms involved in the protective effects of Rab27a dependent exosomes are associated with down‐regulating NOX2 and Cleaved caspase‐3 expression.

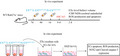

## 1 INTRODUCTION

Ischemic stroke (IS) accounts for 80% of stroke and has become the leading cause of death and disability worldwide. So far, the pathophysiology of IS has not been well understood, and there is lack of efficient therapeutic strategy for IS. Currently, efforts should be made to reduce neurovascular injury in the acute phase of IS. The multicellular crosstalk within brain is critical to the maintaining brain vascular homeostasis and functions.^1^ The signaling processes between brain parenchymal cells and cerebral endothelial contribute to vascular remodeling in response to ischemic injury.^2^, ^3^ The increased vascular function is critical for neurological function recovery after stroke.^4^ Therefore, understanding the mechanisms of these intercellular interactions is pivotal in developing novel therapeutic strategies for acute IS.

Apart from the traditional models of cellular interactions, such as paracrine signaling and direct cell–cell contacts,^5^ an increasing number of studies have demonstrated that exosomes (EXs) secreted from various cell types can mediate local and systemic cell–cell interaction without direct cell–cell contact.^6^ EXs are nano‐sized vesicles which could regulate target cell functions by transferring biological cargos, such as lipids, RNAs, and proteins.^6^, ^7^ They are generated as intraluminal vesicles (ILVs) in the lumen of endosomes and secreted by the fusion of mutivesicular bodies (MVBs) with the plasma membrane.^8^ The processes of EX production are complex and involve multiple regulators, such as endosomal sorting complexes required for transport (ESCRT) proteins (e.g., HRS and Tsg101), tetraspanins (e.g. CD9 and CD81), and Rab GTPases (e.g. Rab31 and Rab27),^9^, ^10^, ^11^, ^12^ and the underlying mechanisms remain largely unclear. In the brain, EXs derived from all types of brain cells are functional by mediating intercellular communication.^13^, ^14^ For example, astrocyte‐released EXs could transport antioxidants and energy substrates to neurons for exerting neuroprotective effect.^15^ EXs derived from hypoxia‐stimulated pericytes can enhance the angiogenesis in brain.^16^ EXs from inflammation activated brain ECs have been shown to activate the neighboring cells in central nervous systems for tissue repair.^13^ These studies imply the important roles of brain EXs in regulating vascular functions and brain homeostasis under physiological and pathological conditions. However, the molecular mechanisms that regulating brain EXs release and their effects on cerebrovascular and brain ischemic injury are unknown.

To explore the role and mechanism of brain EXs in IS, we analyzed the gene expression profile in the brains of IS mice and identified Rab27a as one of the prominently up‐regulated gene in response to tMCAO, paralleling with the increase in EXs secretion. Rab proteins are small (21–25 kDa) monomeric GTPase/GTP‐binding proteins that play critical roles in the regulation of cell membrane‐trafficking events, such as endocytosis and exocytosis.^17^, ^18^ Rab27 has been proved to implicate in EXs secretion in Hela cells through regulating MVBs docking at the plasma membrane.^12^ Moreover, Rab27‐dependent EXs production contributed to hematopoietic homeostasis and prevention of aberrant chronic inflammation.^19^ A recent study in a liver ischemic injury model has suggested that Rab27a mediated liver EXs secretion contributes to hepatic ischemia/reperfusion injury.^20^ It is unclear whether Rab27a‐mediated brain endogenous EXs secretion might be involved in regulating cerebral vascular functions and subsequent brain acute ischemic injury.

In this study, we first analyzed brain gene expression and EXs secretion in wide type (WT) mice in response to tMCAO and identified that Rab27a was the responsible gene for the activation of brain EX release in ischemic situation. Then, we determined the role of Rab27a in IS by measuring neurological deficit score (NDS), infarct volume, cerebral blood flow (CBF), and cerebral microvascular density (cMVD) in Rab27a knockout (Rab27a^−/−^) mice. To further investigate the effects of Rab27a‐dependent EXs, EXs isolated from Rab27a^−/−^ or WT mouse brain tissue were pre‐administrated into cerebral ischemic Rab27a^−/−^ mice. Since vascular ECs apoptosis and oxidative stress‐induced vascular injury and integrity disruption play a pivotal role in the initiation and development of IS, the roles of these EXs on ECs oxidative stress and apoptosis were further determined in both in vitro and in vivo ischemic models. The underlying mechanisms were investigated by analyzing the NOX2 and cleaved caspase‐3 expression which have been shown to play critical roles in ischemia‐induced ROS overproduction and apoptosis in ECs.^21^, ^22^, ^23^

## 2 MATERIALS AND METHODS

### 2.1 RNA extraction and mRNA‐seq analysis

Total RNAs were extracted from brain tissues of peri‐infarct area of one IS and one similar brain region of sham group mice using TRIzol (Invitrogen, USA) according to manufacturer's instructions. Validation was done by QRT‐PCR in ten mice with IS and ten sham group mice. Subsequently, total RNAs were assessed by electrophoresis on a denaturing agarose gel and quantified by NanoDrop spectrophotometer (NanoDrop, USA). 2 μg total RNAs were used for stranded RNA sequencing library preparation using TruSeq Stranded mRNA LT Sample Pre Kit (Illumina) following the manufacturer's instruction. Then, RT‐PCR reactions were performed with Phusion high‐fidelity DNA polymerase, index (X) primer, and universal PCR primers. Finally, the mRNA products were fragmented and purified using the AgencourtAMPure XPPCR (AMPure XP) purification beads and library qualities were evaluated on an Agilent 2100 Bioanalyzer system. The RNA libraries were sequenced on an Illumina Hiseq 4000 platform, and 150‐bp paired‐end reads were generated. Data processing of raw reads was quality checked by using fastqc v0.10.1 (http://www.bioinformatics. babraham.ac.uk/projects/fastqc/). Clean reads were mapped to the reference genome by using HISAT2 v2.0.4. The mapped reads of each sample were assembled by using StringTie v1.3.1 in a reference‐based approach.^24^ DEGene analysis was performed using the DESeq2 R package. DESeq2 provides statistical routines for determining differential expression based on Gene expression data using a model based on the negative binomial distribution. The resulting P‐values were adjusted using the Benjamini–Hochberg approach for controlling the false discovery rate (FDR). Gene with an adjusted *p*‐value of <0.05 (FDR <0.05) by DESeq and with a fold change of ≥2 (|log2 fold change| ≥1) were considered to be differentially expressed.

### 2.2 Quantitative RT‐PCR

Total RNAs were extracted from brain tissues of tMCAO and sham group mice with TRIzol reagent (Invitrogen, USA), and cDNA was synthesized with the RevertAid First Strand cDNA Synthesis kit (Thermo Scientific). Quantitative RT‐PCR (qRT‐PCR) was performed in triplicate with SYBR Premix Ex Taq (Takara, Japan). The primers for the genes were synthesized by Sangon Biotech (Shanghai, China), as follows: 5′‐ AGACCAGAGGGCAGTGAAAGAGG‐3′ (forward) and 5′‐ CACCGCTCCATCCGCTTCATG‐3′(reverse) for Rab27a;5′‐ ACTGCCACAGACCTCTCCAACC‐3′ and 5′‐CTACTGCCACCCACCTCTCCTC‐3′ for Ykt6; 5′‐CCATTGGGGCTGCCTTTCTAACC‐3′ (forward) and 5′‐CAACTATGGCTGCTTGTGCTCCTC‐3′ (reverse) forRab5a; 5′‐GCACGGAAGACAGAAAGAGACG‐3′ and 5′‐TGATAGGCTGGAGGAGGGATTG‐3′ for Pdcp6id; 5′‐CGGGCTAAGAATTGGGTGAAGGAG‐3′ and 5′‐TCATCTGCATAGGCTTGTGCTTCC‐3′ for Rab5c; and 5′‐GAAGGGCTCATGACCACAGTCCAT‐3′ and 5′‐TCATTGTCGTACCAGGAAATGAGCTT‐3′ for GAPDH. GAPDH was used to normalize mRNA expression. The relative quantification of the gene expression was calculated by the 2^‐ΔΔCT^ method.

### 2.3 Isolation of brain EXs

EXs were isolated from brain tissue of WT (EX^WT^) and Rab27a^−/−^ (EX^Rab27a−/−^) mice as a previous study described.^25^ Briefly, the right (ischemic) hemibrains were separated and finely minced with a small sharp scissors in 100 μl papain solution (20 units/ml). Brain samples in solution were pipette into a 15‐ml conical tube containing 3.5 ml papain solution and incubated at 37°C for 20 min to dissociate the tissue. Then, the solutions were centrifuged 300 *g* for 10 min to discard brain cells, and the supernatants were collected and centrifuged at 2000 g for 20 min to remove cells and debris. The collected supernatants were ultra‐centrifuged at 20,000 *g* for 90 min and then at 100,000 g for 2 h to pellet tissue EXs. The pelleted EXs were resuspended with filtered phosphate‐buffered saline (PBS), and aliquot for nanoparticle tracking analysis (NTA) and transmission electron microscopy (TEM). Additionally, the EXs‐specific markers including CD63 and TSG101 were measured by western blot analysis.

### 2.4 Efficiency of EXs purification analysis

A known amount (5 × 10^8^ particles) of brain EXs was added into 1 ml of filtered PBS. The EXs/PBS mixture was centrifuged at 100,000 *g* for 2 h at 4°C to pellet brain EXs. The pellets were resuspended with filtered PBS and incubated with rabbit anti‐CD63 for 2 h in a reaction volume, followed by incubation with goat anti‐rabbit IgG conjugated with Q‐dot® 655 (1:350 dilution; Life Technologies) for 90 min at RT. Then, filtered PBS was added to the EXs suspension (incubated with CD63) to give a final volume of 700 μl and subsequently analyzed by fluorescence NTA. The efficiency of EXs purification rate was calculated as: the number of CD63+ EXs divided by the total number of EXs.

### 2.5 Animal study

The male adult (8–10 weeks and weight 22–24 g) C57BL/ 6 WT and Rab27a knockout (Rab27a^−/−^) mice were purchased from Cyagen Biosciences Company (Guangzhou, China) and housed in the Animal Care Facility at the Guangdong Medical University. The mice were maintained in a pathogen‐free environment with free access to food and water on a 12 h light/dark cycle before and after surgery. Transient middle cerebral artery occlusion (tMCAO) surgery was conducted as we previously described^26^ for inducing IS. In brief, mice were anesthetized with 2.5% isoflurane inhalation, and body temperature was maintained through a thermostat‐controlled heating pad. The left common carotid artery, external carotid arteries (ECA), and internal carotid artery (ICA) were isolated and ligated. A 2.0 cm length of monofilament nylon suture was inserted from the right ECA into the lumen of ICA, and then advanced until resistance was felt. Reperfusion was initiated by withdrawal of the monofilament after 90 min occlusion. Surgeries were finished and animals were placed back into their cages. Pain and discomfort were minimized by an initial injection of buprenorphine (0.1 mg/kg, sc) and carperofen (5 mg/kg, sc) followed with another carperofen injection every 24 h. After tMCAO, mice were transferred to a heating pad for 12 h to keep warm. All experimental procedures were approved by the Laboratory Animal Care and Use Committees at Guangdong Medical University. The animal data reporting in this study has followed the ARRIVE 2.0 guidelines.^27^

At least, 14 tMCAO surgeries were included to ensure we had 12 IS mice per group. At 48 h after tMCAO surgery, NDS and CBF were determined in WT and Rab27a^−/−^ mice. Then, brain samples were harvested for analysis of tissue EXs in the peri‐infarct area, or for histological analyses of infarct volume and cMVD. To evaluate the role of Rab27a^−/−^ dependent EXs secretion in acute IS, Rab27a^−/−^ mice were included and randomly divided into four groups: (1) sham group: Mice underwent the same procedure of tMCAO surgery, excepting that the monofilament was inserted. (2) vehicle group: Mice were pre‐infused with 100 μl PBS twice (3 days apart) before tMCAO surgery (24 h after second injection). (3) EX^WT^ group: Mice were injected via tail vein with EXs isolated from WT mice brain (EX^WT^) (1 × 10^11^ EXs/ 100 μl) thrice (3 days apart) before tMCAO surgery (24 h after third injection). (4) L‐EX^Rab27a−/−^ group: EXs isolated from brain tissue of Rab27a^−/−^ mice (EX^Rab27a−/−^) (4 × 10^10^ EXs/ 100 μl) were injected thrice (3 days apart) before tMCAO surgery (24 h after third injection). (5) H‐EX^Rab27a−/−^ group: EX^Rab27a−/−^ (1 × 10^11^ EXs/ 100 μl) were injected thrice (3 days apart) before tMCAO surgery (24 h after third injection). The dose of EX^WT^ was chosen based on previous publications on EXs infusion for treating IS or brain injury in mice.^28^ In addition, we tested the dose effect of EX^Rab27a−/−^ in our study. At 48 h after tMCAO, the mice were used for various measurements, including CBF, NDS, cMVD, infarct volume, cerebral EC apoptosis, and ROS production.

### 2.6 Structured illumination microscopy

The levels of EXs in brain tissue were detected by three‐dimensional structured illumination microscopy (3D‐SIM) as a previous study described with modification.^11^ The brains were dissected from mice and frozen in liquid nitrogen and then cut into 20‐μm‐thick sections. The mice brain tissue sections were incubated with Rabbit anti‐CD63 (1:100, abcam) and Mouse monoclonal anti‐CD31 (1:100; Invitrogen) at 4 °C overnight. Subsequently, the slices were incubated with the following secondary antibodies for 1 h at room temperature: Alexa fluor647 labeled donkey anti‐rabbit secondary antibody (1:500, abcam) for CD63, and goat anti‐mouse IgG H&L (Alexa Fluor® 488) (1:500, abcam) for CD31. Cellular nuclear was stained with DAPI (1:1000, abcam) for 7 min at room temperature. After wash with washing buffer, the brain sections were collected for image analysis using a Nikon super‐resolution microscope equipped with a 100 × silicone oil immersion objective. Images were captured using Nikon NIS‐Elements and taken using Z‐stacks with step sizes of 0.12 μm. To quantify the EXs numbers in brain sections, CD63‐positive particles in 5 random fields were averaged for individual slice by using NIS‐Elements Ar with N‐SIM imaging and analysis module command with these options: Size (μm), 0.05–0.2; Circularity, 0.01–1.

### 2.7 Nanoparticle tracking analysis

The concentration and size distribution of EXs were measured by NTA as we previously described.^29^ In brief, suspended EXs were diluted in 1 ml PBS and applied to NanoSight (NS300) to automatically measure the average diameter and concentration.

### 2.8 Transmission electron microscopy

For evaluating the quality of isolated EXs by electron microscopy, 10 μl suspended EXs were pipette onto carbon‐coated copper grids. After the sample was dry, micrographs were taken with a calibrated magnification of 100,000‐fold by a TEM.^28^

### 2.9 Detection of EXs merging with ECs in the peri‐infarct area

The PKH26 (Sigma) labeled EXs (1 × 10^11^ EXs/ 100 μl) were injected into Rab27a^−/−^ mice via tail vein according to our previous report with minor modification.^28^ Briefly, the PKH26 labeled EXs were washed with 1 × PBS and pelleted by ultracentrifuge at 100, 000 g for 90 mins to clear the free PKH26 before they were administrated into mice via tail vein. EXs‐free PBS which has received the same treatment as EXs solution was set as vehicle control. After 24 h of EXs infusion, mice were subjected to tMCAO surgery. The brains were isolated 24 h after tMCAO and frozen in liquid nitrogen and then cut into sections (20‐μm‐thick). Brain sections were fixed with 4% paraformaldehyde, permeabilized with 0.5% Triton X‐100 for 10 min, and incubated with Mouse monoclonal anti‐CD31 (1:100; Invitrogen) for cerebral ECs at 4 °C overnight and the sections were then incubated with goat anti‐mouse IgG H&L (Alexa Fluor® 488) (1:500, abcam) secondary antibody for 1 h. DAPI dye (358 nm) was used to stain cell nucleus. After rinsing with wash solution, the sections were observed under a confocal microscope (Olympus Corporation, Japan) for determining the merger of EXs with ECs.

### 2.10 Detection of ROS production in cerebral ECs in the peri‐infarct Area

As we previously reported,^30^ ROS production in cerebral ECs was measured using DHE (Beyotime, Molecular Probes) fluoromicrography based on manufacturer's instructions. Rab27a^−/−^ mice were pre‐infused with PBS, EX^WT^, L‐EX^Rab27a−/−^, or H‐EX^Rab27a−/−^ and then subjected to tMCAO surgery. 48 h later, mice were sacrificed and DHE (2 μM) was superfused cortically for 60 min by intracardiac injection. At the end of the perfusion, the mice brains were quickly removed and frozen in −80 °C, cut into 20 μm, and incubated with Mouse monoclonal anti‐CD31 (1:100; Invitrogen) for microvessels (1:100, abcam, USA) at 4 °C overnight, following with incubation of goat anti‐mouse IgG H&L (Alexa Fluor® 488) (1:200，abcam, USA) for 60 min. The nuclei were counterstained with DAPI for 7 min at room temperature. The ROS‐dependent vascular fluorescence was observed by confocal microscopy. The percentage of DHE‐positive ECs of each slice was calculated from the average value of 5 randomly selected fields in per‐infarct area.

### 2.11 Detection of cerebral EC apoptosis

The cerebral ECs apoptosis was detected by TUNEL assay kit (Beyotime) according to the manufacturer's instructions. In brief, the mice brain tissue sections (20 μm) were incubated with Mouse monoclonal anti‐CD31 (1:100; Invitrogen) for cerebral ECs at 4 °C overnight, after incubated with Cy3 goat anti‐mouse secondary antibody, the slices were further incubated with TUNEL working solution (Beyotime, china) for 60 min at 37°C. The nuclei were counterstained with DAPI for 7 min at room temperature. The slices were observed under a confocal microscopy. The labeled ECs (TUNEL+CD31+) were considered as apoptotic cerebral ECs. The labeled cells in the peri‐infarct area of each section were counted in 5 random fields by an investigator who was unaware of grouping information.

### 2.12 Measurements of cerebral blood flow and microvascular density

The CBF of mice was measured using PeriCam PSI System (Perimed, Sweden) as we previously described.^30^ Briefly, Rab27a^−/−^ mice were pre‐infused with PBS or various EXs and then subjected to tMCAO surgery. After 48 h, mice were anesthetized and placed on a stereotaxic apparatus. A crossing skin incision was made on the head to expose the whole skull. PeriCam PSI System scanning was performed on the intact skull for approximately 1 min. The relative CBF was calculated using the formula: CBF of ipsilateral side/CBF of contralateral side×100%. The cerebral microvascular density (cMVD) was measured as we previously described by using CD31 (1:50; Invitrogen) staining.^26^ The microvascular was counted when its length is twice its width. The mean density of cMVD from six sequential brain sections of the individual mouse was calculated by an investigator who was unaware of grouping and expressed as numbers/mm^2^.

### 2.13 Measurements of neurological deficits and infarct volume

The infarct volume and neurological deficit scores (NDS) were measured by 2% 2,3,5‐triphenyltetranzolium chloride (TTC) staining and 5‐point scale method as we previously reported.^26^ All tests were performed by an investigator who was unaware of the grouping information.

### 2.14 Culture of primary brain ECs

The microvascular endothelial cells (ECs) of mouse brain were isolated and cultured from Rab27a^−/−^ mice based on a previous report with minor modification.^31^ Briefly, the mice were humanely killed by inhalation of excess CO_2_ and cleansed three times with 75% alcohol. The brain and its cerebral hemispheres were removed and placed in a petri dish containing a sterile PBS solution. The surface blood vessels, pia mater, and cerebral medulla were removed from the cortex. The harvested tissue was cut into pieces, homogenized into suspensions, and filtered through an 80 μm mesh screen. The collected tissues were then filtered through a 200‐μm mesh screen. The oversize residue was collected and centrifuged at 1000 rpm for 5 min at 4°C. The resulting precipitate was collected and digested with 0.2% type II collagenase (Sigma‐Aldrich, St. Louis, MO, United States) in phosphate‐buffered saline at 37°C for 30 min and then centrifuge at 1000 *g* for 5 min at 4°C. At last, the microvessel pellets were resuspended in 10 ml endothelial cell culture medium (10% FBS, 30 μg/mL ECGS, 15 U/ml heparin, 325 μg/ml glutathione, 1 μl/mL 2‐mecaptoethanol, 100 U/ml penicillin, and 100 μg/ml streptomycin, sigma) and plated on rat tail collagen 1 coated six‐well cell culture plates at 37°C with 5% CO_2_ in air. The growth of other types of cells and impurities was reduced through the differential adhesion method. Cells were washed twice with PBS and digested with 1 ml of trypsin–EDTA (0.25% trypsin with 0.25% EDTA) for 3–4 min until the majority of cells contract to round. Full medium was added to end the digestion and cells were shook off and suspended with full medium, and seeded into culture flasks pre‐coated with rat tail collagen 1. Cells were incubated at 37°C in 5% CO_2_. The medium was replaced every 3 days. In addition, we added experiment to detect the purity of isolated ECs by the endothelial cell marker CD31. The cells were incubated with Mouse monoclonal anti‐CD31 (1:100; Invitrogen) at 4°C overnight, and incubated with goat anti‐mouse IgG H&L (Alexa Fluor® 488) (1:500, abcam) for 1 h at room temperature. Cellular nuclear was stained with DAPI (1:1000, abcam) for 7 min at room temperature. The percentage of CD31+ ECs was examined under a fluorescence microscope (Leica, TCS SP5II, Germany).

### 2.15 Co‐culture assay of EXs with ECs

EXs were labeled with PKH26, a red fluorescence cell membrane dye, according to the manufacturer's protocol. Briefly, EXs (50 μg/ml) were labeled with PKH26 (2 μM) at room temperature (RT) for 5 min. An equal volume of 1% bovine serum albumin (BSA) was added to stop staining. The PKH26‐labeled EXs were co‐culture (37°C, 5% CO_2_) with ECs for 12 h. EXs‐free PBS which has received the same treatment as EXs solution was set as vehicle control. Then, cells were washed with PBS and incubated with fluorescein isothiocyanate (FITC)‐conjugated anti‐beta Actin antibody (abcam, 1:100) for 1 h at room temperature. The incorporation of EXs into ECs was examined under a fluorescence microscope (Leica, TCS SP5II, Germany).

To explore the effects of Rab27a‐dependent EXs secretion on ischemia‐induced EC injury. ECs were co‐cultured with culture medium or incubated with EX^WT^, L‐EX^Rab27a−/−^, or H‐EX^Rab27a−/−^ for 24 h before subjected to oxygen and glucose deprivation (OGD). ECs co‐cultured with culture medium under normoxic conditions for 24 h was set as control group. OGD experiments were performed as we previously described.^26^ Briefly, the ECs were cultured in glucose‐free Dulbecco's modified Eagle medium (life technology, USA) and incubated in a hypoxia incubator (1% O_2_, 5% CO_2_, and 94% N_2_; Thermo Fisher Scientific, USA) and maintained at 37°C for 6 h. Cells were then substituted with fresh normal culture medium and incubated in a standard 5% CO2 incubator for 24 h. After that, cells were collected for ROS, apoptosis, and signaling pathway protein expression analysis.

### 2.16 ROS production analysis in cultured ECs

Intracellular ROS level in ECs was measured by dihydroethidium (DHE; Beyotime) staining followed with immunofluorescence analysis as we reported previously.^30^ Briefly, ECs were pre‐incubated with EX^WT^, L‐EX^Rab27a−/−^, H‐EX^Rab27a−/−^, or culture medium for 24 h and then subjected to OGD treatment followed with incubation of DHE solutions (5 μM) for 2 h at 37°C. The fluorescence intensity of ROS in each slice was detected under a fluorescence microscope and calculated from the average value of 5 randomly selected fields by an investigator who was unaware of grouping. The measurement was carried out in triplicate for calculating the average value.

### 2.17 Apoptosis analysis

Cell apoptosis was analyzed by using Annexin V‐PE/7‐AAD apoptosis detection kit (BD Biosciences) according to the manufacturer’ instructions. Briefly, after EXs incubation and OGD as described above, ECs were fixed and stained with Annexin V‐PE and 7‐AAD solution followed by flow cytometry analysis. The measurement was carried out in triplicate and the average value was calculated.

### 2.18 Western blotting analysis

The proteins of ECs were extracted with cell lysis buffer (Applygen Technologies company, China) supplemented with protease inhibitor tablet (Thermo scientific, USA). Protein lysates were electrophoresed trough SDS‐PAGE gels and transferred onto PVDF membranes. The membranes were blocked with 5% non‐fat milk for 1 h and incubated with primary antibodies against β‐actin (1:1000, EarthOx, San Francisco, CA, USA), cleaved caspase‐3 (1:1000, CST, USA), Nox (1:1000, Abcam, USA), TSG101 (1:400, Abcam), CD63 (1:400, Abcam) at 4°C overnight. Membranes were then incubated with HRP‐conjugated anti‐mouse or ant‐rabbit IgG (1:40,000; EarthOx, San Francisco, CA, USA) for 1 h at room temperature. Blots were developed with ECL solution (Amersham, Sweden).

### 2.19 Statistical analysis

The data of NDS were expressed as median (range). All other data are expressed as mean ± SEM (standard error of the mean). Kolmogorov–Smirnov (KS) test was used to examine the data normality. Comparisons for two groups were analyzed by independent *t*‐tests. GraphPad Prism 7 software was used for analyzing the data. Multiple comparisons between or among groups were analyzed by the Kruskal–Wallis test followed by a Tukey post hoc test (SPSS 25, USA). The parametric data including CBF, infarct volume, cell ROS production and apoptosis, and effects of brain EXs treatment were assessed using one‐ or two‐way ANOVA, followed by the Tukey test. For all measurements, a *p* < 0.05 was considered statistic significant.

## 3 RESULTS

### 3.1 Rab27a expression and EXs level were increased in the peri‐infarct area of ischemic brain

**FIGURE 1:**
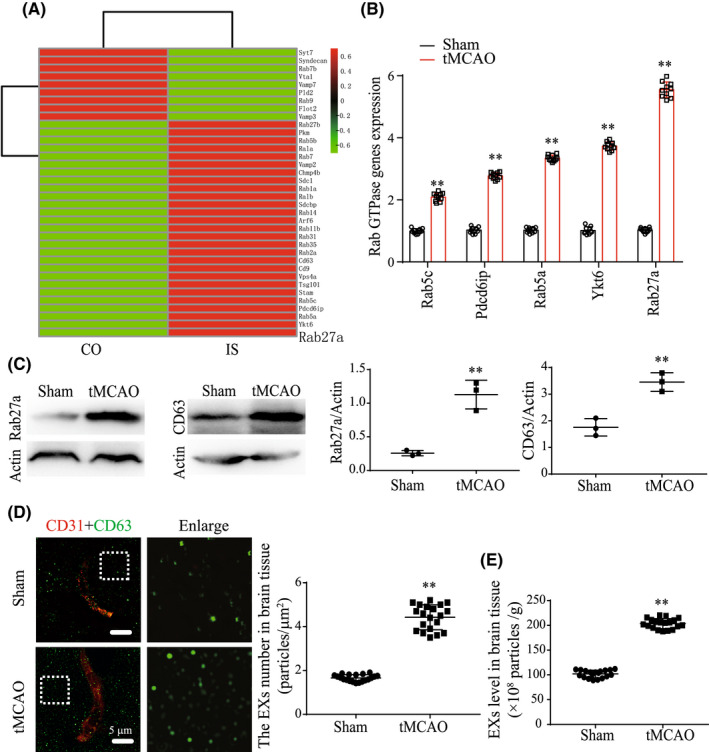
Ischemia increases Rab27a expression and brain EXs secretion. (A) Heat map of proteins involved in EXs biogenesis or secretion by mRNA sequence in brain tissue of control mouse and IS mouse. (B) Comparison of the top five elevated genes including Rab5a, Pdcd6ip, Ykt6, Rab27a, Rab5c by QRT‐PCR. Data were expressed as mean ± SEM (*n* = 3 mice/group). (C) Rab27a and CD63 expression in brain of control mouse and IS mouse were detected by Western blotting. Data were expressed as mean ± SEM (*n* = 3 mice/group). (D) The level of brain EXs were detected by structured illumination microscopy in WT mice after sham or tMCAO surgery. EXs (CD63, green), cerebral vessels (CD31, red). Data were expressed as mean ± SEM, *n* = 20 fields from 4 mice per group. (E) The level of brain EXs were detected by NTA assay in WT mice after sham or tMCAO surgery. Data were expressed as mean ± SEM, *n* = 20 fields from 4 mice per group. *p < 0.05, **p < 0.01 compared with the sham group

To explore the molecular mechanisms by which ischemia regulates EXs secretion, we performed mRNA‐seq analysis to screen the differentially expressed genes in the peri‐infarct area of tMCAO mice compared with the sham group mice and analyzed the gene profiles related to EXs biogenesis and secretion. A heat map performed on the differentially expressed mRNAs is shown in Figure 1A. Based on this mRNA profiling data, we selected the top five up‐regulated mRNAs including: Rab5c, Pdcd6ip, Rab5a, Ykt6, and Rab27a, and validated their expression by using qRT‐PCR. As shown in Figure 1B, Rab27a was the most significantly upregulated gene related to EXs generation in peri‐infarct area of tMCAO mice (*T* = 19.91, *p*_tMCAO vs sham_ <0.0001). We further verified that the expression of Rab27a by western blot analysis (Figure 1C: *T* = 6.964, *p*_tMCAO vs sham_ = 0.0022). Additionally, we observed that the expression of EXs‐specific marker CD63 was substantially increased in peri‐infarct area of brain tissue (*T* = 6.152, *p*_tMCAO vs sham_ = 0.0035; Figure 1C). According to the data of super‐resolution microscopy and NTA analysis, the level of EXs in peri‐infarct area of brain tissue was substantially increased at day 2 after tMCAO in WT mice (Figure 1D: *T* = 21.07, *p*_tMCAO vs sham_ <0.0001; Figure 1E: *T* = 8.41, P_tMCAO vs sham_ <0.0001). These data indicated that the Rab27a and EXs levels in brain tissue were up‐regulated in response to ischemic injury.

### 3.2 Rab27a deficiency decreased EXs secretion and aggravated the brain ischemic injury

**FIGURE 2:**
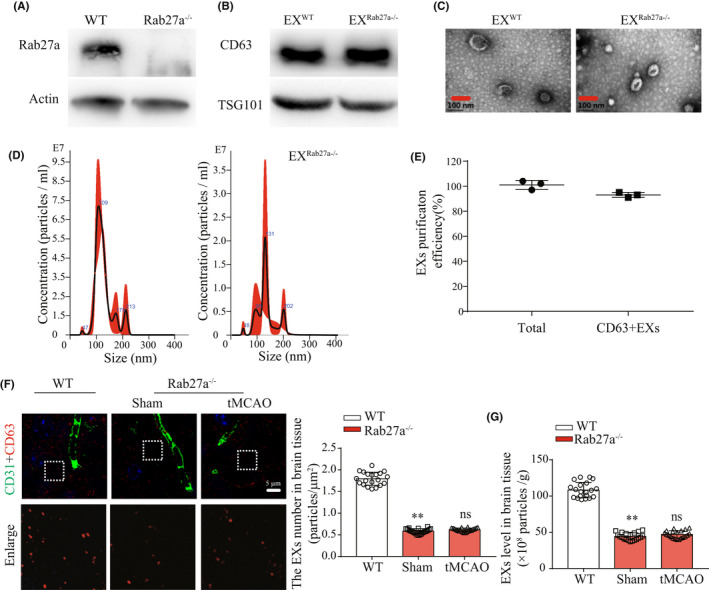
Levels of brain EXs were significantly reduced in Rab27a^−/−^ mice. (A) Rab27a expression in brain tissue of WT mice and Rab27a^−/−^ mice was measured by Western blotting. (B) EXs‐specific marker CD63 and TSG101 were detected by Western blotting. (C) TEM was used to detect the size and morphology of EX^WT^ or EX^Rab27a−/−^. (D) The concentration and size of EXs from brain tissue of WT mice (EX^WT^) or Rab27a^−/−^ mice (EX^Rab27a−/−^) were detected by NTA. (E) The purification efficiency of CD63+ EXs in the total brain vesicles; data were expressed as mean ± SEM, *n* = 3 mice per group. (F) The level of brain EXs in WT mice and Rab27a^−/−^ mice under normal or IS condition were observed using structured illumination microscopy. EXs (CD63, red), cerebral vessels (CD31, green). (G) The level of brain EXs in WT mice and Rab27a^−/−^ mice under normal or IS condition were detected by using NTA. *p < 0.05, **p < 0.01 compared with the WT group; ^ns^ *p*>0.05 compared to the sham group; all data were expressed as mean ± SEM, *n* = 20 fields from 4 mice per group

Rab27a was totally knocked out in the brain tissue of Rab27a^−/−^ mice (vs WT; Figure 2A). We isolated EXs from brain tissue of WT and Rab27a^−/−^ mice. Western blot analysis confirmed the expression of EX special markers CD63 and TSG101 (Figure 2B). NTA and TEM analyses of brain tissue EXs showed that EX^WT^ and EX^Rab27a−/−^ were similar in size with diameter of 100 ± 50 nm (Figure 2C,D). Fluorescence NTA analysis showed that higher percentages (≥90%) of brain EXs were recovered by our isolation and purification methods (Figure 2E), indicating the majority of element we collected was EXs through our isolation and purification method. Moreover, we found that the level of EXs in brain tissue of Rab27a^−/−^ mice was decreased by about 60% as measured by using super‐resolution microscopy (Figure 2F: *T* = 33.34, *p*_Rab27a−/− vs WT_ <0.0001) and NTA methods (Figure 2G: *T* = 24.32, *p*_Rab27a−/− vs WT_ <0.0001). Interestingly, tMCAO did not significantly increase the level of EXs in peri‐infarct area of Rab27a^−/−^ mice as measured by super‐resolution microscopy (Figure 2F: *T* = 1.96, *p*_Rab27a−/− vs WT_ = 0.058) and NTA methods (Figure 2G: *T* = 1.73, *p*_Rab27a−/− vs WT_ = 0.092), suggesting that ischemia‐induced brain EXs increase depends on the Rab27a pathway.

**FIGURE 3:**
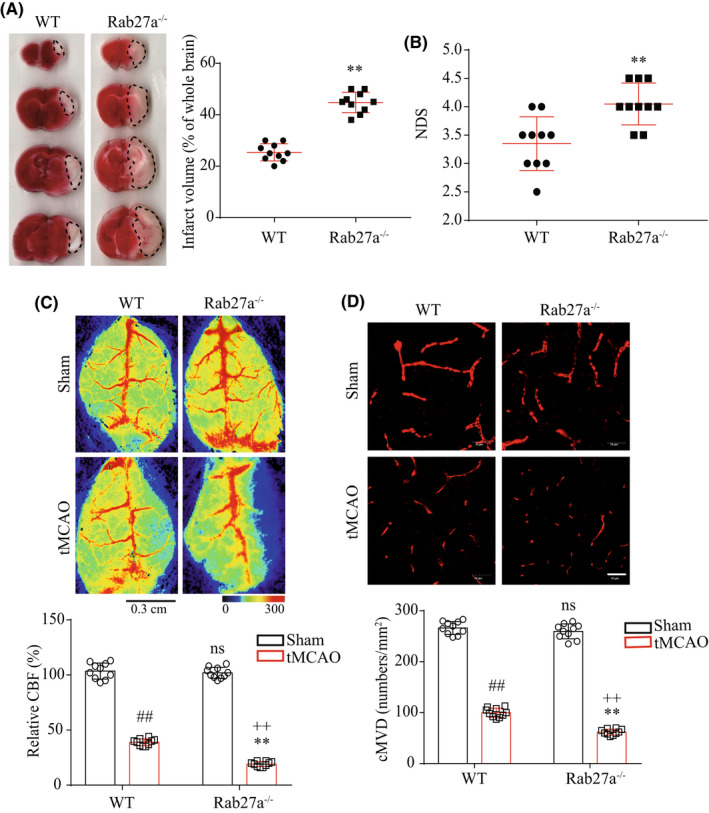
Rab27a^−/−^ mice displayed aggravated cerebral ischemic injury. (A) Representative images and summarized data showing the infarct volume in WT mice and Rab27a^−/−^ mice after tMCAO surgery. (B) Summarized data showing the NDS of WT mice and Rab27a^−/−^ mice after tMCAO surgery. (C) Representative images and summarized data showing the CBF in WT mice and Rab27a^−/−^ mice after tMCAO surgery. (D) Representative images and summarized data showing the cMVD in WT mice and Rab27a^−/−^ mice after tMCAO surgery. ^##^ *p* < 0.01 or ^ns^ *p*>0.05 compared to the sham group in WT mice; *p < 0.05, **p < 0.01 compared with the tMCAO group in WT mice; ^++^ *p* < 0.01 compared with the sham group in Rab27a^−/−^ mice; ^ns^ *p* > 0.05 compared with the sham group in WT mice. All data were expressed as mean ± SEM (*n* = 10 mice/group)

After 48 h of tMCAO surgery, the cerebral injury was assessed by measuring the infarct volume, NDS, cMVD, and CBF. As shown in Figure 3, the infarct volume (Figure 3A: *T* = 11.78, *p*_Rab27a−/− vs WT_ <0.0001) and NDS (Figure 3B: *T* = 3.68, *p*_Rab27a−/− vs WT_ = 0.0018) were more severe in Rab27a^−/−^ mice. In addition, we also observed that the CBF (Figure 3C: *T* = 16.36, *p*_Rab27a−/− vs WT_ <0.0001) and cMVD (Figure 3D: *T* = 11.17, *p*_Rab27a−/− vs WT_ <0.0001) was significantly lower in Rab27a^−/−^ mice after tMCAO surgery, suggesting that Rab27a deficient compromises the collateral microcirculation in the brain. These data indicated that Rab27a^−/−^ mice are more sensitive to cerebral ischemic injury.

### 3.3 Systemic administration of EXs increased the level of EXs in peri‐infarct area of IS mice

**FIGURE 4:**
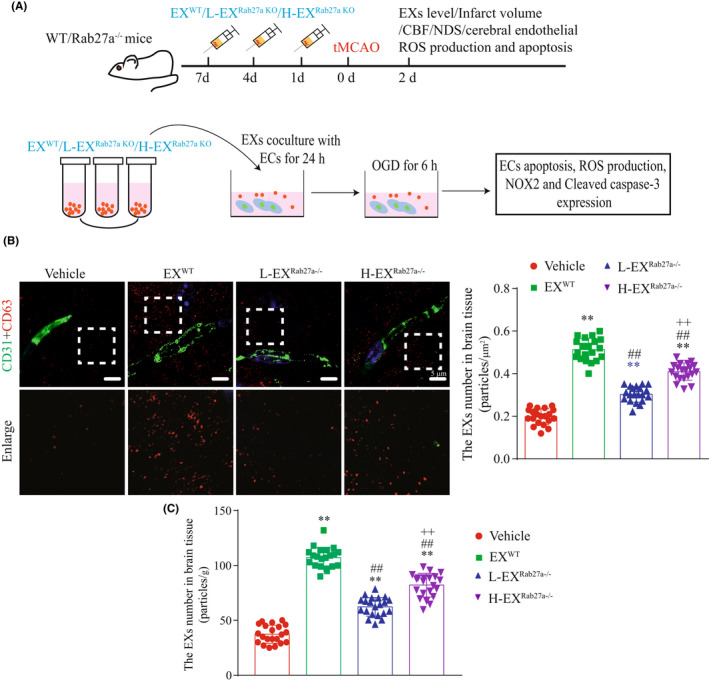
Pre‐infusion of EXs increased the brain EXs level in Rab27a^−/−^ mice. (A) Schematic depicting the workflow for the in vivo and in vitro experiments. (B) Immunofluorescence of CD31 (green) and CD63 (red) in the peri‐infarct area of Rab27a^−/−^ mice using super‐resolution microscopy. Scale bar: 5 μm. (C) EXs levels in the peri‐infarct brain tissues in different EX infusion groups measured by super‐resolution microscopy and NTA. *p < 0.05, **p < 0.01 compared with the vehicle group; ^#^ *p* < 0.05, ^##^ *p* < 0.01 compared with the EX^WT^ group; ^+^ *p* < 0.05, ^++^ *p* < 0.01 compared with the L‐EX^Rab27a−/−^ group. All data were expressed as mean ± SEM, *n* = 20 fields from 4 mice per group

To determine the efficiency of systemic infusion of EXs on increasing the level of EXs in the peri‐infarct area of Rab27a^−/−^ mice, PBS, EX^WT^, L‐EX^Rab27a−/−^ or H‐EX^Rab27a−/−^ were injected via tail vein injection (Figure 4A), and the EXs level in peri‐infarct area of brain tissue in Rab27a^−/−^ mice were measured by super‐resolution microscopy and NTA analysis. We observed that the level of EXs in brain tissue of Rab27a^−/−^ mice were significantly increased when pre‐infusion of EX^WT^ as measured by using super‐resolution microscopy (Figure 4B: *T* = 21.83, *p*_EX(WT) vs Vehicle_ <0.0001) and NTA methods (Figure 4C: *T* = 25.7, *p*_EX(WT) vs Vehicle_ <0.0001). Compared to EX^WT^ treatment, pre‐infusion of H‐EX^Rab27a−/−^ exerted attenuated effects on increasing EXs level as measured by using super‐resolution microscopy (Figure 4B: *T* = 6.94, *p*_H‐EX(Rab27a−/−) vs EX(WT)_ < 0.0001) and NTA methods (Figure 4C: *T* = 8.01, *p*_H‐EX(Rab27a−/−) vs EX(WT)_ < 0.0001), and L‐EX^Rab27a−/−^ displayed even less effects as measured by using super‐resolution microscopy (Figure 4B: *T* = 8.77, *p*_L‐EX(Rab27a−/−) vs H‐EX(Rab27a−/−)_ < 0.0001) and NTA methods (Figure 4C: *T* = 6.61, *p*_L‐EX(Rab27a−/−) vs H‐EX(Rab27a−/−)_ < 0.0001). Our results indicated systemic infusion of EXs could increase the local level of EXs in the peri‐infarct area of ischemic brain and that Rab27a plays a critical role in restoring EXs level in peri‐infarct area.

### 3.4 Rab27a deficiency decreased the effects of EXs on reducing cerebral vascular ROS overproduction and EC apoptosis in IS mice

**FIGURE 5:**
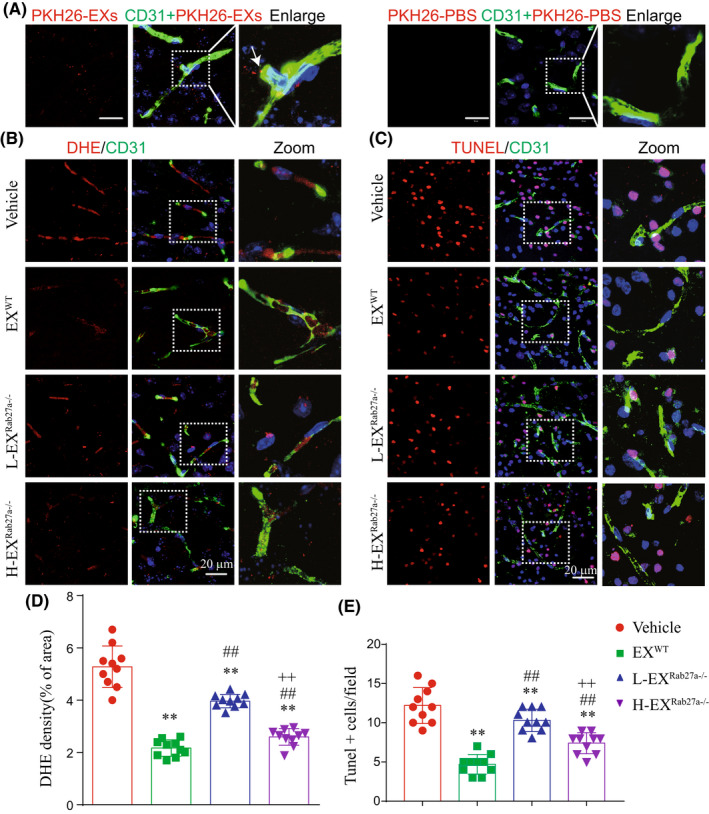
Pre‐infusion of EXs decreased the cerebral vascular ROS overproduction and apoptosis in peri‐infarct area of Rab27a^−/−^ mice. (A) Immunofluorescence of EX^WT^ (PKH26, red) or PKH26‐PBS merged with ECs (CD31, green). Scale bar: 20 μm. (B) Immunofluorescence of ROS (DHE, red) with ECs (CD31, green) in peri‐infarct area of Rab27a^−/−^ mice. Scale bar: 20 μm. (C) Immunofluorescence of apoptotic cells (Tunnel staining, red) with ECs (CD31, green) in peri‐infarct area of Rab27a^−/−^ mice. Scale bar: 20 μm. (D) Summarized data showing the cerebral vascular ROS production in the peri‐infarct area of Rab27a^−/−^ mice. (E) Summarized data showing the cerebral ECs apoptosis in the peri‐infarct area of Rab27a^−/−^ mice. *p < 0.05, **p < 0.01 compared to the vehicle group; ^#^ *p* < 0.05, ^##^ *p* < 0.01 compared with the EX^WT^ group; ^+^ *p* < 0.05, ^++^ *p* < 0.01 compared with the L‐EX^Rab27a−/−^ group. All data were expressed as mean ± SEM (*n* = 10 mice/group)

EX^WT^ labeled with PKH26 were pre‐infused into tMCAO operated Rab27a^−/−^ mice. The fluorescent of PKH26 labeled EX^WT^ was observed in cerebral microvessels of Rab27a^−/−^ mice brain at 48 h after tMCAO (Figure 5A). We further detected the effects of EX^WT^, L‐EX^Rab27a−/−^, or H‐EX^Rab27a−/−^ on ischemia‐induced cerebral vascular ROS production and apoptosis in Rab27a^−/−^ mice. We found that infusion of EX^WT^ significantly decreased the cerebral vascular ROS overproduction (Figure 5B,D: *T* = 11.49, *p*_EX(WT) vs Vehicle_ <0.0001) and EC apoptosis (Figure 5C,E: *T* = 9.06, *p*_EX(WT) vs Vehicle_ <0.0001) in peri‐infarct area of Rab27a^−/−^ mice. Compared with EX^WT^ treatment, pre‐infusion of H‐EX^Rab27a−/−^ exerted attenuated effects on decreasing the cerebral vascular ROS overproduction (Figure 5B,D: *T* = 2.99, *p*_H‐EX(Rab27a−/−) vs EX(WT)_ = 0.0079) and EC apoptosis (Figure 5C,E: *T* = 4.64, *p*_H‐EX(Rab27a−/−) vs EX(WT)_ = 0.0002), and L‐EX^Rab27a−/−^ displayed even less effects on decreasing the cerebral vascular ROS overproduction (Figure 5B,D: *T* = 10.57, *p*_L‐EX(Rab27a−/−) vs H‐EX(Rab27a−/−)_ < 0.0001) and EC apoptosis (Figure 5C,E: *T* = 4.68, *p*_L‐EX(Rab27a−/−) vs H‐EX(Rab27a−/−)_ = 0.0002). Our results indicated that Rab27a mediated EXs secretion was important for protecting cerebral microvessels from ischemia‐induced ROS overproduction and EC apoptosis, and the effects were dose‐dependent.

### 3.5 Rab27a deficiency decreased the effects of EXs on protecting mice from cerebral ischemic injury

**FIGURE 6:**
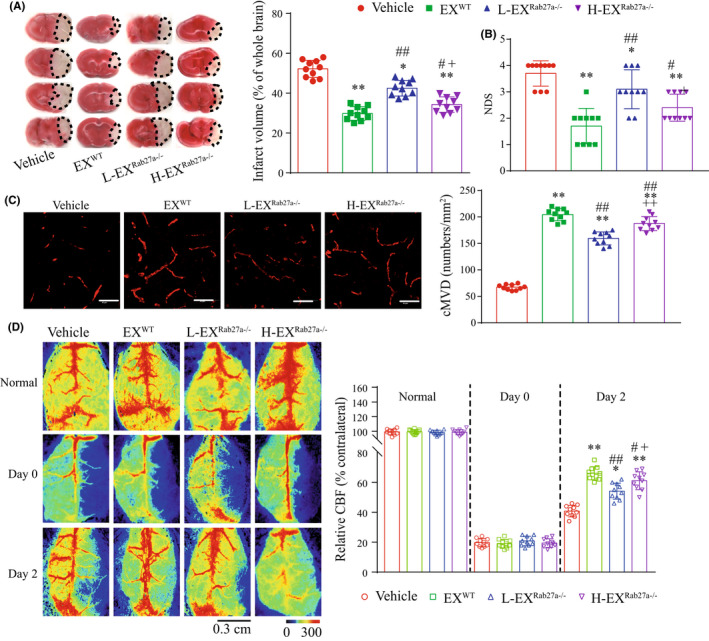
Pre‐infusion of EXs protected Rab27a^−/−^ mice from ischemic injury. (A) Representative images and summarized data showing the infarct volume in tMCAO operated Rab27a^−/−^ mice. (B) Summarized data showing the NDS of Rab27a^−/−^ mice after tMCAO surgery. (C) Immunofluorescence of cMVD (CD31, green) in peri‐infarct area of Rab27a^−/−^ mice. Scale bar: 40 μm. Summarized data showing the cMVD in the peri‐infarct area of Rab27a^−/−^ mice. (D) Representative images and summarized data showing the CBF in tMCAO operated Rab27a^−/−^ mice. *p < 0.05, **p < 0.01 compared with the vehicle group; ^#^ *p* < 0.05, ^##^ *p* < 0.01 compared with the EX^WT^ group; ^+^ *p* < 0.05, ^++^ *p* < 0.01 compared with the L‐EX^Rab27a−/−^ group. All data were expressed as mean ± SEM (*n* = 10mice/group)

The effects of pre‐infusion of EX^WT^, L‐EX^Rab27a−/−^, or H‐EX^Rab27a−/−^ on cerebral injury were further explored by analyzing the cMVD, CBF, infarct volume, and NDS in tMCAO operated Rab27a^−/−^ mice. We found that infusion of EX^WT^ significantly increased the cMVD (Figure 6C: *T* = 34.68, *p*_EX(WT) vs Vehicle_ <0.0001), while decreased the infarct volume (Figure 6A: *T* = 12.86, *p*_ex(WT) vs Vehicle_ <0.0001) and NDS (Figure 6B: *T* = 6.45, *p*_EX(WT) vs Vehicle_ <0.0001) in tMCAO Rab27a^−/−^ mice. Compared to EX^WT^ treatment, pre‐infusion of H‐EX^Rab27a−/−^ exerted attenuated effects on increasing the cMVD (Figure 6C: *T* = 3.07, *p*_H‐EX(Rab27a−/−) vs EX(WT)_ = 0.0066), and decreasing the infarct volume (Figure 6A: *T* = 2.75, *p*_H‐EX(Rab27a−/−) vs EX(WT)_ = 0.013) and NDS (Figure 6B: *T* = 2.61, *p*_EX(WT) vs Vehicle_ = 0.018), and L‐EX^Rab27a−/−^ displayed even less protective effects in increasing the cMVD (Figure 6C: *T* = 4.92, *p*_L‐EX(Rab27a−/−) vs H‐EX(Rab27a−/−)_ = 0.0001) and decreasing the infarct volume (Figure 6A: *T* = 4.69, *p*_L‐EX(Rab27a−/−) vs H‐EX(Rab27a−/−)_ = 0.0002) and NDS (Figure 6B: *T* = 2.46, *p*_L‐EX(Rab27a−/−) vs H‐EX(Rab27a−/−)_ = 0.024).

As shown in Figure 6D, the data on day 0, which the CBF was detected immediately after tMCAO, indicated that all the mice received an equal amount of ischemic insult. On day 2, the CBF was significantly increased after EX^WT^ administration (Figure 6D: *T* = 13.71, *p*_EX(WT) vs Vehicle_ <0.0001). Compared with EX^WT^ treatment, pre‐infusion of H‐EX^Rab27a−/−^ exerted attenuated effects on increasing the CBF (Figure 6D: *T* = 2.14, *p*_H‐EX(Rab27a−/−) vs EX(WT)_ = 0.046), and L‐EX^Rab27a−/−^ displayed even less protective effects in increasing the CBF (Figure 6D: *T* = 2.76, *p*_L‐EX(Rab27a−/−) vs H‐EX(Rab27a−/−)_ = 0.013). Our results indicated that Rab27a play a pivotal role in protecting cerebral microvessels and brain from ischemic injury by regulating the release amount of EXs as well as the functions of individual EX.

### 3.6 Rab27a‐dependent EXs protected ECs from OGD‐induced ROS overproduction, apoptosis, and upregulation of NOX2 and Caspase‐3

**FIGURE 7:**
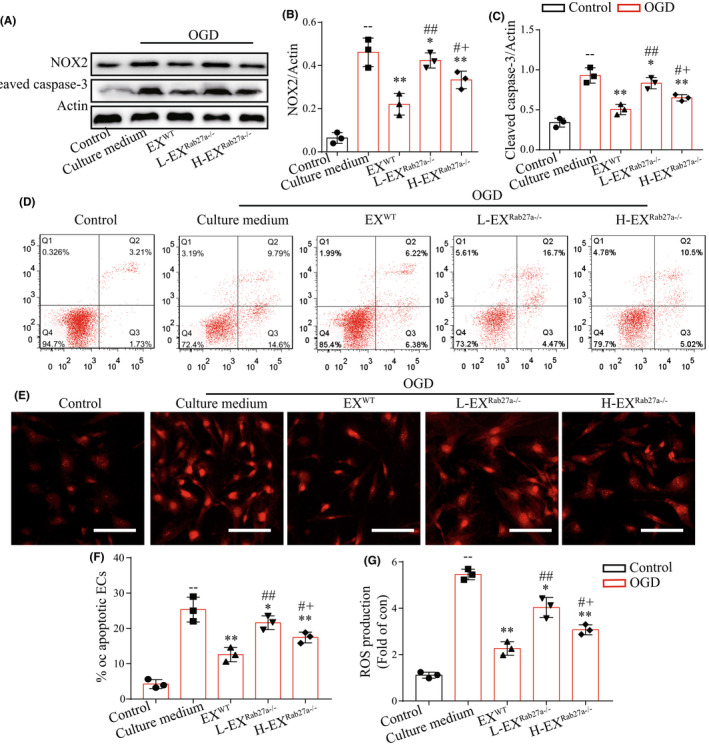
Pre‐incubation of EXs decreased ECs ROS overproduction and apoptosis under OGD condition. (A–C) Representative bands and summarized data showing the protein level of NOX and cleaved caspase‐3 in ECs. (D, F) Representative images and summarized data showing the apoptosis of ECs pre‐incubated with various EXs. (E, G) Representative images and summarized data showing the ROS production in ECs pre‐incubated with various EXs. ^−^ *p* < 0.05, ^− −^ *p* < 0.01 compared with the control group; *p < 0.05, **p < 0.01 compared with the culture medium group; ^#^ *p* < 0.05, ^##^ *p* < 0.01 compared with the EX^WT^ group; ^+^ *p* < 0.05, ^++^ *p* < 0.01 compared with the L‐EX^Rab27a−/−^ group. All data were expressed as mean ± SEM (*n* = 3/group)

Primary ECs were cultured from Rab27a^−/−^ mice, and we observed a high purity of up to 95% CD31+ ECs by our isolation and purification methods (Additional file 2: Figure S1). To explore the role of brain EXs in ischemic ECs, we conducted OGD experiments in cultured brain microvascular ECs. After co‐cultured of ECs with PKH26‐labeled EXs for 24 h, we observed EXs in the cytoplasm of ECs (Figure S2). As shown in Figure 7, pre‐incubation of EX^WT^ significantly decreased the NOX2 expression (Figure 7A,B: *T* = 5.05, *p*_EX(WT) vs culture medium_ = 0.0073) and ROS production (Figure 7E,G: *T* = 14.93, *p*_EX(WT) vs culture medium_ = 0.0001) in OGD‐injured ECs. Compared to EX^WT^ treatment, pre‐incubation of H‐EX^Rab27a−/−^ exerted attenuated effects on reducing NOX2 expression (Figure 7A,B: *T* = 3.05, *p*_H‐EX(Rab27a−/−) vs EX(WT)_ = 0.038) and ROS production (Figure 7E,G: *T* = 3.88, *p*_H‐EX(Rab27a−/−) vs EX(WT)_ = 0.018), and L‐EX^Rab27a−/−^ displayed even less effects in decreasing NOX2 expression (Figure 7A,B: *T* = 2.91, *p*_L‐EX(Rab27a−/−) vs H‐EX(Rab27a−/−)_ = 0.044) and ROS production (Figure 7E,G: *T* = 3.41, *p*_L‐EX(Rab27a−/−) vs H‐EX(Rab27a−/−)_ = 0.027).

According to the flow cytometry analysis, we found that pre‐incubation of EX^WT^ significantly decreased the cleaved caspase‐3 expression (Figure 7A,C: *T* = 6.38, *p*_EX(WT) vs culture medium_ = 0.0031) and apoptosis (Figure 7D,F: *T* = 5.47, *p*_EX(WT) vs culture medium_ = 0.0054) in OGD‐injured ECs. Compared with EX^WT^ treatment, pre‐incubation of H‐EX^Rab27a−/−^ exerted decreased effects on reducing the cleaved caspase‐3 expression (Figure 7A,C: *T* = 3.30, *p*_H‐EX(Rab27a−/−) vs EX(WT)_ = 0.03) and apoptosis (Figure 7D,F: *T* = 3.36, *p*_H‐EX(Rab27a−/−) vs EX(WT)_ = 0.028), and L‐EX^Rab27a−/−^ displayed even less effects in decreasing the cleaved caspase‐3 (Figure 7A,C: *T* = 3.93, *p*_L‐EX(Rab27a−/−) vs H‐EX(Rab27a−/−)_ = 0.017) and apoptosis (Figure 7D,F: *T* = 2.92, *p*_L‐EX(Rab27a−/−) vs H‐EX(Rab27a−/−)_ = 0.043). Taken together, our results indicated that Rab27a‐dependent EXs secretion from brain tissue could protect ECs from OGD induced ROS overproduction and apoptosis via regulating NOX2/ROS and cleaved caspase‐3 pathway, and the effects were dose‐dependent.

## 4 DISCUSSION

The major findings of this study are that ischemia could promote Rab27a expression and EXs secretion in peri‐infarct area of mouse brain, and that Rab27a^−/−^ mice were more sensitive to ischemia‐induced cerebral vascular injury. In the tMCAO model of Rab27a^−/−^ mice, pre‐infusion of brain EXs could promote vascular functions and reduce brain injury, and protect cerebral ECs from oxidative stress and apoptosis. In OGD/R model of ECs, brain EXs could decrease oxidative stress and apoptosis via inhibiting NOX2 and Caspase‐3 expression. These beneficial effects were decreased in brain EX^Rab27a−/−^.

The multicellular crosstalk within brain could be critical to maintain the homeostasis of vascular microcirculation and the normal function of brain microenvironment.^2^ Previous studies by ours and others demonstrate that EXs play a key role in mediating intercellular communication between ECs, neurons and astrocytes, and in maintaining neurovascular homeostasis and functions under physiological or ischemic conditions.^13^, ^32^, ^33^ However, the regulation of EXs production in brain is largely unknown and the role of brain tissue EXs in IS remains unclear. In the present study, by using structured illumination microscopy (SIM) and NTA techniques, we firstly observed that EXs production in peri‐infarct area of acute IS mouse was obviously increased. Our finding is supported by a previous study demonstrating that the EXs level in ischemic liver tissue was significantly increased.^20^ Recently, SIM with high resolution (X,Y axis: 115 nm, Z axis:269 nm) has been widely used to detect the cellular biological structures, such as lysosomes,^11^ mitochondrias,^34^ and extracellular vesicles^35^ in cultured cells. In the present study, we firstly used the 3D‐SIM to analyze the EXs in peri‐infarct area of brain tissue. To investigate the possible genes responsible for the brain EXs biogenesis and secretion, we analyzed the differential expressed genes which may be involved in EXs biogenesis and secretion in ischemic brain.^36^, ^37^, ^38^ Our data showed that Rab27a was the mostly elevated protein, indicating that Rab27a might involve in the EXs biogenesis and secretion of ischemic brain. Rab27a is an important member of Rab protein family. Different Rab isoforms have been shown to play diverse functional roles in regulating the generation and function of EXs.^39^ For example, Rab22a has been reported to mediate EXs secretion of breast cancer cell and could promote cell proliferation and migration.^40^ Rab35 has been proved to regulate oligodendroglia EXs secretion, which may play functional role in central nervous system^41^ Recently, Rab27‐dependent bone marrow EXs has been demonstrated to inhibit chronic inflammation in bacterial LPS‐treated Rab27a and Rab27b double knockout mice.^19^ Rab27a and Rab27b have been shown to regulate EX secretion by affecting transport or docking of MVBs to the target plasma membrane in Hela cells.^12^

To explore the effects of Rab27a‐dependent EXs production on cerebral ischemic injury, we carried out experiments utilizing the Rab27a‐deficient mouse model. As expected, the brain EXs level was obviously reduced in Rab27a^−/−^ mice, which was in line with previous studies demonstrating that Rab27a can control EX secretion in Hela cell lines and in mice liver.^12^, ^20^ However, tMCAO did not significantly increase the level of EXs in peri‐infarct area of Rab27a^−/−^ mice. Our finding suggested that Rab27a was essential in brain EXs secretion in ischemic condition. Rescue experiment that builds Rab27a overexpression in brain tissue of Rab27a^−/−^ mice can further verify the role of Rab27a in controlling brain EXs secretion and functions. Moreover, we found that Rab27a^−/−^ mice had decreased cMVD and CBF at the acute stage of IS. Studies have reported that decreased cMVD and CBF after stroke were associated with increased infarct volume and NDS.^33^, ^42^ To our expectation, the infarct volume and NDS were increased in Rab27a^−/−^ mice. These indicated that Rab27a contribute to brain EXs secretion and might play important roles in protecting cerebrovascular and brain ischemic injury. We further pre‐infused brain EXs from WT or Rab27a^−/−^ mice into tMCAO Rab27a^−/−^ mice and found that both types of brain EXs could increase EXs level in mice, suggesting that the administration of ectogenic EXs could restore brain EXs level under ischemic condition. Interestingly, EX^WT^ mice were more effective than EX^Rab27a−/−^ mice on increasing brain EXs level. These differences may be attributed to the high ability of Rab27a‐dependent EXs to cross the blood‐brain barrier. There are reports demonstrated that various EX membrane proteins, such as adhesive molecules, membrane transferring proteins contributed to promote EXs entry into the brain.^43^ Further study is needed to clarify whether Rab27a‐dependent EXs are enriched with these proteins. What is more, pre‐infusion of EXs from WT or Rab27a^−/−^ mice could merge into cerebral vessels and increase cMVD and CBF of IS mice. To be noted, EXs from WT mice were more effective than EXs from Rab27a^−/−^ mice on promoting cMVD and CBF. There are reports from ours and other researches demonstrated that the functions of EXs on target cells are highly correlated with their cargos.^33^, ^44^ Therefore, we supposed that Rab27a‐dependent EXs may enriched with vasoprotective bioactivators, which need further investigation. Our findings first imply the critical roles of Rab27a not only in regulating EXs secretion but also in modulating the cargos of EXs in brain tissue. Further study showed that EX^WT^ could more effectively reduce the infarct volume and NDS of IS mice than EX^Rab27a−/−^.

Since cMVD and CBF are important for maintaining neurovascular function after IS,^28^, ^33^ we presumed that Rab27a‐dependent EXs could inhibit brain ischemic injury by protecting vascular functions. In addition, EX^Rab27a−/−^ with high concentration were more effective than with low concentration on regulating EXs level and neurovascular functions investigated in this study, demonstrating that their effects were in a dose‐dependent manner. Our data indicated that the endogenously produced brain EXs play a critical role in protecting cerebral vessels and brain from acute ischemic injury, and Rab27a was important for their beneficial effects. In recent years, EXs have been considered as fundamental mechanism of communication between tissue cells and play important roles in microenvironment homeostasis in liver,^20^ hematopoietic system,^19^ central nerves system.^13^ Our data add new evidence to the protective effect of brain EXs in maintaining brain and vascular homeostasis. In the present study, we focus on the role of Rab27a in regulating brain EXs secretion and in cerebral vascular damage from ischemic injury. We did not clarify the effects of Rab27a on the secretion of EXs from specific brain cells, such as endothelial cells, astrocytes, and neurons on ischemic stroke. Of note, Rab27a has been found to express in a wide range of brain cells, including neurons,^45^ microglial cells,^46^ endothelial progenitor cells,^47^ and astrocytes.^48^ EXs derived from some brain cells under ischemic condition have been reported to be increased and associated with neurovascular function recovery after stroke. Ischemic pre‐condition has shown to promote astrocyte EXs release and contribute to axonal outgrowth and functional remodeling after ischemic stroke.^44^ Our previous studies demonstrated that the levels of EXs derived from endothelial progenitor cells (EPC‐EXs) were significantly increased in plasma and brain tissue after ischemic stroke^49^, ^50^ and were negatively correlated with infarct volume and cell apoptosis, while positively correlated with microvessel density.^50^ Therefore, Rab27a might be implicated in promoting EPCs or astrocytes EXs releasing after stroke and these EXs might contribute to protect against cerebral ischemic injury, which will be detected in our future research work. Meanwhile, we observed that Rab27a knockout did not completely abolish the generation of brain EXs, suggesting that there are other proteins implicated in mediating brain EXs section and their therapeutic effects, which need further investigation.

Cerebral vascular oxidative stress and apoptosis is the initiation of various CNS diseases, such as ischemic stroke,^51^ vascular dementia,^52^ and Alzheimer's disease.^53^ It has been proved by us and others that EXs derived from ECs, stem cells, or astrocytes could protect neurovascular cells from ischemia‐induced oxidative stress and apoptosis.^33^, ^44^, ^54^ In this study, we determined the role of Rab27a in the effects of brain EXs on ischemia‐induced endothelial oxidative stress and apoptosis. We found that the infusion of EXs from WT mice and Rab27a^−/−^ could reduce ischemia‐induced cerebral EC oxidative stress and apoptosis in vitro and in vivo. The effects of EX^Rab27a−/−^ were reduced when compared with EX^WT^. Recently, there are evidence from us and others showing that exosomal cargos, such as ACE2,^55^ miR‐132‐3p,^33^ TIMP2,^56^ etc can enhance the effects of EXs on ischemia‐induced EC apoptosis and oxidative stress. Thus, Rab27a‐dependent EXs may enriched with these cargos, which need further investigation. In addition, we observed that H‐EX^Rab27a−/−^ displayed better efficacy than L‐ EX^Rab27a−/−^ in vitro and in vivo, suggesting that the protective effects of Rab27a‐mediated brain EXs section are dose‐dependent. Overall, these findings indicated that Rab27a‐mediated brain EXs secretion might exert their protective effects on ischemic injured cerebral vessels via reducing cerebral EC oxidative stress and apoptosis. In the present study, we only used adult male mice to study the role of Rab27a‐dependent EXs in protecting against cerebral ischemic injury. Sex has been shown to be an important factor involved in cerebral ischemic injury and functional outcome after stroke.^57^, ^58^, ^59^ There is evidence demonstrated that female‐APOE ε4 carriers showed a faster reduction in cerebral blood flow than that of the male‐APOE ε4 carriers.^59^ The female and male individuals were also shown to suffer different pathological damage from stroke, including differential oxidative stress, inflammation, and apoptosis.^57^ Recently, Partha K Chandra., et al reported the sex disparities in gene expression and canonical pathway in brain microvessels, which could explain at least in part the stroke outcome differences in male versus female subjects.^60^ Therefore, further studies are needed to investigate the effects of Rab27a‐dependent brain EXs on protecting against cerebral ischemic injury in mice of different sexes.

To further clarify the underlying mechanism involved in the protective effects of Rab27a‐dependent brain EXs in OGD‐induced oxidative stress in brain ECs, we detected the NOX2 expression, which has been proved to be important in regulating ROS production.^61^ We found that EXs could significantly decrease NOX2 expression and EXs lacking Rab27a were less effective. Our findings indicate that the antioxidant effect of Rab27a in brain EXs via the NOX2/ROS pathway. Meanwhile, we also found that EX^WT^ or EX^Rab27a−/−^ pre‐treatment could remarkably decreased cleaved caspase‐3 expression in OGD‐injured ECs, and EX^WT^ were more effective than H‐EX^Rab27a−/−^. Cleaved casepase‐3 is a typically pro‐apoptosis protein.^62^ Thus, these data indicated that Rab27a dependent brain EXs could protect ECs from OGD‐induced oxidative stress and apoptosis via inhibiting NOX2 and cleaved caspase‐3 expression. Not surprisingly, the effects of these EXs on reducing NOX2 and cleaved caspase‐3 were dose‐dependent, which were correspondence with their effects on regulating EC oxidative stress and apoptosis.

Although our findings indicate that Rab27a‐dependent brain EXs secretion is important to maintain cerebral vascular homeostasis and prevention of ischemic injury via reducing vascular EC oxidative stress and apoptosis, it remains to be clarified which cell origin of the EXs plays a major role. Novel mouse strains with cell‐specific knockout and overexpression of Rab27a gene are expected to address this question. Our recent studies have demonstrated that the antioxidative stress and antiapoptosis effects of EXs are highly depend on their cargos, such as protein and microRNAs.^33^, ^55^ Therefore, further studies are needed to explore the functional contents in Rab27a dependent brain EXs and their relationship with NOX2 and cleaved caspase‐3.

## 5 CONCLUSIONS

In conclusion, our findings suggest that Rab27a is a major protein controlling brain EXs secretion at both physiological and ischemic conditions, which is essential for maintaining cerebral vascular homeostasis and preventing brain ischemic injury via reducing oxidative stress and apoptosis in acute stage of ischemic stroke.

## AUTHOR CONTRIBUTIONS

XM, JZ, YW, JL, YS, JL, SL, YC, and QP performed experiments; XM, JZ, YW, and QP wrote the manuscript; XM, QP, and YC contributed to manuscript preparation and developed the concepts and designed the study; all authors discussed the results, analyzed data, and commented on the manuscript and also read and approved the final manuscript.

## CONFLICT OF INTEREST

The authors declare that they have no competing interests.

## CONSENT FOR PUBLICATION

Not applicable.

## Supporting information


**Fig S1** Characterization of primary ECs. Microscopy images of ECs‐specific marker CD31 (green), scale bars = 30 μm.

**Fig S2** The incorporation of EXs with ECs after co‐culture. Immunofluorescence of EX^WT^ (PKH26, red) or PKH26‐PBS merged with ECs (Actin, green). Scale bar: 30 μm.

## Data Availability

All data generated or analyzed during this study are included in this published article.

## References

[1] Abbott NJ , Friedman A . Overview and introduction: the blood‐brain barrier in health and disease. Epilepsia. 2012;53(Suppl 6):1‐6. 10.1111/j.1528-1167.2012.03696.x PMC3625728 23134489

[2] Hermann DM , ElAli A . The abluminal endothelial membrane in neurovascular remodeling in health and disease. Sci Signal. 2012;5(236):re4. 22871611 10.1126/scisignal.2002886

[3] Abbott NJ . Astrocyte‐endothelial interactions and blood‐brain barrier permeability. J Anat. 2002;200(6):629‐638. 12162730 10.1046/j.1469-7580.2002.00064.x PMC1570746

[4] Venkat P , Cui C , Chopp M , et al. MiR‐126 mediates brain endothelial cell exosome treatment‐induced neurorestorative effects after stroke in type 2 diabetes mellitus mice. Stroke. 2019;50(10):2865‐2874. 31394992 10.1161/STROKEAHA.119.025371 PMC6756941

[5] Goodenough DA , Goliger JA , Paul DL . Connexins, connexons, and intercellular communication. Annu Rev Biochem. 1996;65:475‐502. 8811187 10.1146/annurev.bi.65.070196.002355

[6] Lener T , Gimona M , Aigner L , et al. Applying extracellular vesicles based therapeutics in clinical trials ‐ an ISEV position paper. J Extracell Vesicles. 2015;4:30087. 26725829 10.3402/jev.v4.30087 PMC4698466

[7] Kramer‐Albers EM , Hill AF . Extracellular vesicles: interneural shuttles of complex messages. Curr Opin Neurobiol. 2016;39:101‐107. 27183381 10.1016/j.conb.2016.04.016

[8] van Niel G , D'Angelo G , Raposo G . Shedding light on the cell biology of extracellular vesicles. Nat Rev Mol Cell Biol. 2018;19(4):213‐228. 29339798 10.1038/nrm.2017.125

[9] Colombo M , Moita C , van Niel G , et al. Analysis of ESCRT functions in exosome biogenesis, composition and secretion highlights the heterogeneity of extracellular vesicles. J Cell Sci. 2013;126(Pt 24):5553‐5565. 24105262 10.1242/jcs.128868

[10] Sims B , Farrow AL , Williams SD , Bansal A , Krendelchtchikov A , Matthews QL . Tetraspanin blockage reduces exosome‐mediated HIV‐1 entry. Arch Virol. 2018;163(6):1683‐1689. 29429034 10.1007/s00705-018-3737-6 PMC5958159

[11] Wei D , Zhan W , Gao Y , et al. RAB31 marks and controls an ESCRT‐independent exosome pathway. Cell Res. 2021;31(2):157‐177. 32958903 10.1038/s41422-020-00409-1 PMC8027411

[12] Ostrowski M , Carmo NB , Krumeich S , et al. Rab27a and Rab27b control different steps of the exosome secretion pathway. Nat Cell Biol. 2010;12(1):19‐30. sup pp 11–13. 19966785 10.1038/ncb2000

[13] Holm MM , Kaiser J , Schwab ME . Extracellular vesicles: multimodal envoys in neural maintenance and repair. Trends Neurosci. 2018;41(6):360‐372. 29605090 10.1016/j.tins.2018.03.006

[14] Valadi H , Ekstrom K , Bossios A , Sjostrand M , Lee JJ , Lotvall JO . Exosome‐mediated transfer of mRNAs and microRNAs is a novel mechanism of genetic exchange between cells. Nat Cell Biol. 2007;9(6):654‐659. 17486113 10.1038/ncb1596

[15] Ranjit S , Patters BJ , Gerth KA , Haque S , Choudhary S , Kumar S . Potential neuroprotective role of astroglial exosomes against smoking‐induced oxidative stress and HIV‐1 replication in the central nervous system. Expert Opin Ther Targets. 2018;22(8):703‐714. 30015535 10.1080/14728222.2018.1501473

[16] Mayo JN , Bearden SE . Driving the hypoxia‐inducible pathway in human pericytes promotes vascular density in an exosome‐dependent manner. Microcirculation. 2015;22(8):711‐723. 26243428 10.1111/micc.12227 PMC4715585

[17] Barnes S , Kelly ME . Calcium channels at the photoreceptor synapse. Adv Exp Med Biol. 2002;514:465‐476. 12596939 10.1007/978-1-4615-0121-3_28

[18] Hutagalung AH , Novick PJ . Role of Rab GTPases in membrane traffic and cell physiology. Physiol Rev. 2011;91(1):119‐149. 21248164 10.1152/physrev.00059.2009 PMC3710122

[19] Alexander M , Ramstead AG , Bauer KM , et al. Rab27‐dependent exosome production inhibits chronic inflammation and enables acute responses to inflammatory stimuli. J Immunol. 2017;199(10):3559‐3570. 28978688 10.4049/jimmunol.1700904 PMC5821227

[20] Yang MQ , Du Q , Goswami J , et al. Interferon regulatory factor 1‐Rab27a regulated extracellular vesicles promote liver ischemia/reperfusion injury. Hepatology. 2018;67(3):1056‐1070. 29059701 10.1002/hep.29605 PMC5826835

[21] Yang F , Wang Z , Wei X , et al. NLRP3 deficiency ameliorates neurovascular damage in experimental ischemic stroke. J Cereb Blood Flow Metab. 2014;34(4):660‐667. 24424382 10.1038/jcbfm.2013.242 PMC3982086

[22] Chen J , Xiao X , Chen S , et al. Angiotensin‐converting enzyme 2 priming enhances the function of endothelial progenitor cells and their therapeutic efficacy. Hypertension. 2013;61(3):681‐689. 23266545 10.1161/HYPERTENSIONAHA.111.00202 PMC4011714

[23] Wei S , Low SW , Poore CP , et al. Comparison of anti‐oncotic effect of TRPM4 blocking antibody in neuron, astrocyte and vascular endothelial cell under hypoxia. Front Cell Dev Biol. 2020;8:562584. 33195194 10.3389/fcell.2020.562584 PMC7604339

[24] Pertea M , Kim D , Pertea GM , Leek JT , Salzberg SL . Transcript‐level expression analysis of RNA‐seq experiments with HISAT. StringTie and Ballgown Nat Protoc. 2016;11(9):1650‐1667. 27560171 10.1038/nprot.2016.095 PMC5032908

[25] Perez‐Gonzalez R , Gauthier SA , Kumar A , Saito M , Saito M , Levy E . A method for isolation of extracellular vesicles and characterization of exosomes from brain extracellular space. Methods Mol Biol. 2017;1545:139‐151. 27943212 10.1007/978-1-4939-6728-5_10

[26] Pan Q , He C , Liu H , et al. Microvascular endothelial cells‐derived microvesicles imply in ischemic stroke by modulating astrocyte and blood brain barrier function and cerebral blood flow. Mol Brain. 2016;9(1):63. 27267759 10.1186/s13041-016-0243-1 PMC4897950

[27] Percie du Sert N , Hurst V , Ahluwalia A , et al. The ARRIVE guidelines 2.0: updated guidelines for reporting animal research. J Cereb Blood Flow Metab. 2020;40(9):1769‐1777. 32663096 10.1177/0271678X20943823 PMC7430098

[28] Wang J , Chen S , Zhang W , Chen Y , Bihl JC . Exosomes from miRNA‐126‐modified endothelial progenitor cells alleviate brain injury and promote functional recovery after stroke. CNS Neurosci Ther. 2020;26(12):1255‐1265. 33009888 10.1111/cns.13455 PMC7702230

[29] Wang J , Zhong Y , Ma X , et al. Analyses of endothelial cells and endothelial progenitor cells released microvesicles by using microbead and Q‐dot based nanoparticle tracking analysis. Sci Rep. 2016;6:24679. 27094208 10.1038/srep24679 PMC4837394

[30] Zhang H , Pan Q , Xie Z , et al. Implication of MicroRNA503 in brain endothelial cell function and ischemic stroke. Transl Stroke Res. 2020;11(5):1148‐1164. 32285355 10.1007/s12975-020-00794-0

[31] Ruck T , Bittner S , Epping L , Herrmann AM , Meuth SG . Isolation of primary murine brain microvascular endothelial cells. J Vis Exp. 2014;93:e52204. 10.3791/52204 PMC4354020 25489873

[32] Xu B , Zhang Y , Du XF , et al. Neurons secrete miR‐132‐containing exosomes to regulate brain vascular integrity. Cell Res. 2017;27(7):882‐897. 28429770 10.1038/cr.2017.62 PMC5518987

[33] Pan Q , Kuang X , Cai S , et al. miR‐132‐3p priming enhances the effects of mesenchymal stromal cell‐derived exosomes on ameliorating brain ischemic injury. Stem Cell Res Ther. 2020;11(1):260. 32600449 10.1186/s13287-020-01761-0 PMC7322840

[34] Wei Y , Chiang WC , Sumpter R Jr , Mishra P , Levine B . Prohibitin 2 is an inner mitochondrial membrane mitophagy receptor. Cell. 2017;168(1–2):224‐238 e210. 28017329 10.1016/j.cell.2016.11.042 PMC5235968

[35] Choi D , Montermini L , Jeong H , Sharma S , Meehan B , Rak J . Mapping subpopulations of cancer cell‐derived extracellular vesicles and particles by Nano‐flow cytometry. ACS Nano. 2019;13(9):10499‐10511. 31469961 10.1021/acsnano.9b04480

[36] Tiwari N , Wang CC , Brochetta C , et al. VAMP‐8 segregates mast cell‐preformed mediator exocytosis from cytokine trafficking pathways. Blood. 2008;111(7):3665‐3674. 18203950 10.1182/blood-2007-07-103309

[37] Hessvik NP , Llorente A . Current knowledge on exosome biogenesis and release. Cell Mol Life Sci. 2018;75(2):193‐208. 28733901 10.1007/s00018-017-2595-9 PMC5756260

[38] Colombo M , Raposo G , Thery C . Biogenesis, secretion, and intercellular interactions of exosomes and other extracellular vesicles. Annu Rev Cell Dev Biol. 2014;30:255‐289. 25288114 10.1146/annurev-cellbio-101512-122326

[39] Stenmark H . Rab GTPases as coordinators of vesicle traffic. Nat Rev Mol Cell Biol. 2009;10(8):513‐525. 19603039 10.1038/nrm2728

[40] Sun L , He M , Xu N , et al. Regulation of RAB22A by mir‐193b inhibits breast cancer growth and metastasis mediated by exosomes. Int J Oncol. 2018;53(6):2705‐2714. 30272274 10.3892/ijo.2018.4571

[41] Hsu C , Morohashi Y , Yoshimura S , et al. Regulation of exosome secretion by Rab35 and its GTPase‐activating proteins TBC1D10A‐C. J Cell Biol. 2010;189(2):223‐232. 20404108 10.1083/jcb.200911018 PMC2856897

[42] Chen J , Chen S , Chen Y , et al. Circulating endothelial progenitor cells and cellular membrane microparticles in db/db diabetic mouse: possible implications in cerebral ischemic damage. Am J Physiol Endocrinol Metab. 2011;301(1):E62‐E71. 21505143 10.1152/ajpendo.00026.2011 PMC3129837

[43] Jin Q , Wu P , Zhou X , Qian H , Xu W . Extracellular vesicles: novel roles in neurological disorders. Stem Cells Int. 2021;2021:6640836. 33679989 10.1155/2021/6640836 PMC7904361

[44] Hira K , Ueno Y , Tanaka R , et al. Astrocyte‐derived exosomes treated with a Semaphorin 3A inhibitor enhance stroke recovery via prostaglandin D2 synthase. Stroke. 2018;49(10):2483‐2494. 30355116 10.1161/STROKEAHA.118.021272

[45] Song L , Tang S , Han X , et al. KIBRA controls exosome secretion via inhibiting the proteasomal degradation of Rab27a. Nat Commun. 2019;10(1):1639. 30967557 10.1038/s41467-019-09720-x PMC6456494

[46] Wu HY , Chung MC , Wang CC , Huang CH , Liang HJ , Jan TR . Iron oxide nanoparticles suppress the production of IL‐1beta via the secretory lysosomal pathway in murine microglial cells. Part Fibre Toxicol. 2013;10:46. 24047432 10.1186/1743-8977-10-46 PMC3851143

[47] Hassanpour M , Cheraghi O , Brazvan B , et al. Chronic exposure of human endothelial progenitor cells to diabetic condition abolished the regulated kinetics activity of exosomes. Iran J Pharm Res. 2018;17(3):1068‐1080. 30127829 PMC6094433

[48] Loov C , Mitchell CH , Simonsson M , Erlandsson A . Slow degradation in phagocytic astrocytes can be enhanced by lysosomal acidification. Glia. 2015;63(11):1997‐2009. 26095880 10.1002/glia.22873 PMC6728804

[49] Wang J , Guo R , Yang Y , et al. The novel methods for analysis of exosomes released from endothelial cells and endothelial progenitor cells. Stem Cells Int. 2016;2016:2639728. 27118976 10.1155/2016/2639728 PMC4826946

[50] Wang J , Liu H , Chen S , Zhang W , Chen Y , Yang Y . Moderate exercise has beneficial effects on mouse ischemic stroke by enhancing the functions of circulating endothelial progenitor cell‐derived exosomes. Exp Neurol. 2020;330:113325. 32325158 10.1016/j.expneurol.2020.113325 PMC11055452

[51] Liang YQ , Kakino A , Matsuzaka Y , et al. LOX‐1 (lectin‐like oxidized Low‐density lipoprotein Receptor‐1) deletion has protective effects on stroke in the genetic background of stroke‐prone spontaneously hypertensive rat. Stroke. 2020;51(6):1835‐1843. 32397936 10.1161/STROKEAHA.120.029421

[52] Choi DH , Lee J . A mini‐review of the NADPH oxidases in vascular dementia: correlation with NOXs and risk factors for VaD. Int J Mol Sci. 2017;18(11):2500‐2516. 10.3390/ijms18112500 PMC5713465 29165383

[53] Liu Y , Gong Y , Xie W , et al. Microbubbles in combination with focused ultrasound for the delivery of quercetin‐modified sulfur nanoparticles through the blood brain barrier into the brain parenchyma and relief of endoplasmic reticulum stress to treat Alzheimer's disease. Nanoscale. 2020;12(11):6498‐6511. 32154811 10.1039/c9nr09713a

[54] Liu H , Wang J , Chen Y , et al. NPC‐EXs alleviate endothelial oxidative stress and dysfunction through the miR‐210 downstream Nox2 and VEGFR2 pathways. Oxid Med Cell Longev. 2017;2017:9397631. 28630660 10.1155/2017/9397631 PMC5467331

[55] Zhang C , Wang J , Ma X , et al. ACE2‐EPC‐EXs protect ageing ECs against hypoxia/reoxygenation‐induced injury through the miR‐18a/Nox2/ROS pathway. J Cell Mol Med. 2018;22(3):1873‐1882. 29363860 10.1111/jcmm.13471 PMC5824419

[56] Ni J , Liu X , Yin Y , Zhang P , Xu YW , Liu Z . Exosomes derived from TIMP2‐modified human umbilical cord mesenchymal stem cells enhance the repair effect in rat model with myocardial infarction possibly by the Akt/Sfrp2 pathway. Oxid Med Cell Longev. 2019;2019:1958941. 31182988 10.1155/2019/1958941 PMC6512021

[57] Kim TH , Vemuganti R . Effect of sex and age interactions on functional outcome after stroke. CNS Neurosci Ther. 2015;21(4):327‐336. 25404174 10.1111/cns.12346 PMC6495347

[58] Bushnell CD , Chaturvedi S , Gage KR , et al. Sex differences in stroke: challenges and opportunities. J Cereb Blood Flow Metab. 2018;38(12):2179‐2191. 30114967 10.1177/0271678X18793324 PMC6282222

[59] Wang R , Oh JM , Motovylyak A , et al. Impact of sex and APOE epsilon4 on age‐related cerebral perfusion trajectories in cognitively asymptomatic middle‐aged and older adults: a longitudinal study. J Cereb Blood Flow Metab. 2021;41(11):3016‐3027. 34102919 10.1177/0271678X211021313 PMC8545048

[60] Chandra PK , Cikic S , Baddoo MC , et al. Transcriptome analysis reveals sexual disparities in gene expression in rat brain microvessels. J Cereb Blood Flow Metab. 2021;41(9):2311‐2328. 33715494 10.1177/0271678X21999553 PMC8392780

[61] Sirker A , Zhang M , Shah AM . NADPH oxidases in cardiovascular disease: insights from in vivo models and clinical studies. Basic Res Cardiol. 2011;106(5):735‐747. 21598086 10.1007/s00395-011-0190-z PMC3149671

[62] Kluck RM , Bossy‐Wetzel E , Green DR , Newmeyer DD . The release of cytochrome c from mitochondria: a primary site for Bcl‐2 regulation of apoptosis. Science. 1997;275(5303):1132‐1136. 9027315 10.1126/science.275.5303.1132

