# Supplementary figures and images for: Rab27a‐dependent exosomes protect against cerebral ischemic injury by reducing endothelial oxidative stress and apoptosis
Source: CNS Neurosci Ther. 2022 Jun 29;28(10):1596–612. doi: 10.1111/cns.13902 (PMC9437240; doi:10.1111/cns.13902)

DAPI+CD31

Merge

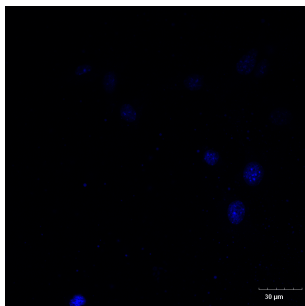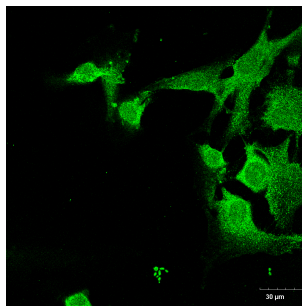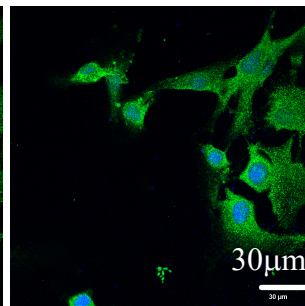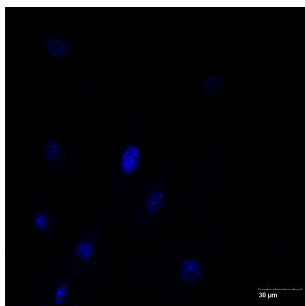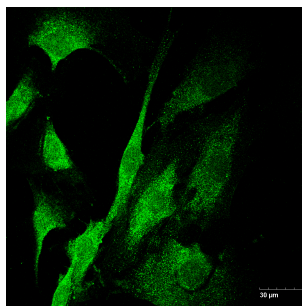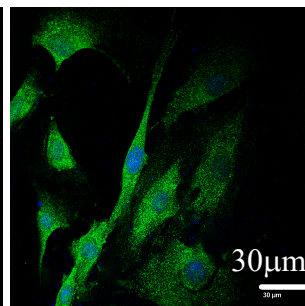

Supplement: Supplementary file 1 — Fig S1 Characterization of primary ECs. Microscopy images of ECs‐specific marker CD31 (green), scale bars = 30 μm. [file CNS-28-1596-s001.pdf]

DAPI

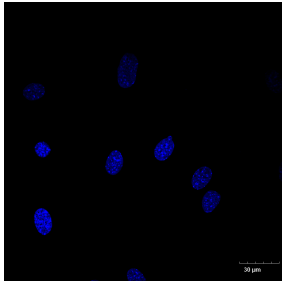

PKH26-PBS

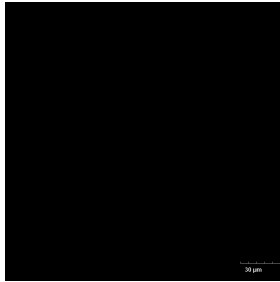

Actin

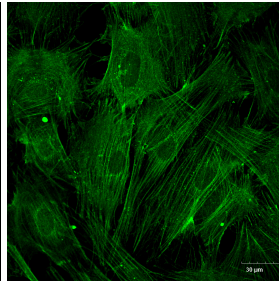

Merge

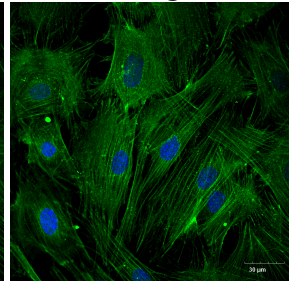

DAPI

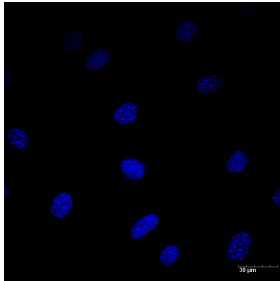

PKH26-EXs

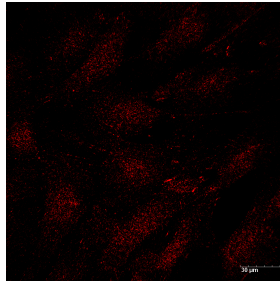

Actin

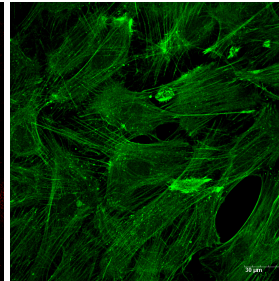

Merge

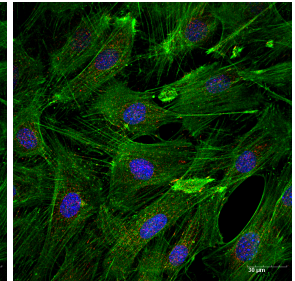

Supplement: Supplementary file 2 — Fig S2 The incorporation of EXs with ECs after co‐culture. Immunofluorescence of EXWT (PKH26, red) or PKH26‐PBS merged with ECs (Actin, green). Scale bar: 30 μm. [file CNS-28-1596-s002.pdf]
